# The Neurobehavioral Profile of Phelan–McDermid Syndrome: Suggestions for Assessment Tools in Light of the 2023 Consensus Guidelines

**DOI:** 10.3390/genes17080923

**Published:** 2026-08-05

**Authors:** Emily Payne, Sara M. Sarasua, Curtis Rogers, Rebekah Martin, Katy Phelan, Laura Beamer, Luigi Boccuto

**Affiliations:** 1 Healthcare Genetics and Genomics Program, School of Nursing, Clemson University, Clemson, SC 29634, USA; emilykp4@gmail.com (E.P.); smsaras@clemson.edu (S.M.S.); rmartin208@outlook.com (R.M.); 2 RCR Genetics, Greenville, SC 29601, USA; crogers126@icloud.com; 3 Greenwood Genetic Center, Greenwood, SC 29646, USA; lbeamer@ggc.org; 4 Genetics Laboratory, Florida Cancer Specialists & Research Institute, Fort Myers, FL 33916, USA; kphelan@flcancer.com

**Keywords:** Phelan–McDermid Syndrome, assessment tool, clinical decision-making, consensus guidelines, behavior, intellectual disability

## Abstract

Objectives and Background: Individuals with Phelan–McDermid Syndrome (PMS) present with a variety of symptoms, including a breadth of behavioral issues. Clinically assessing behavior in PMS remains challenging due to the overabundance of behavioral assessments and the lack of tools validated explicitly for use in individuals with intellectual disability (ID) and neurodevelopmental disorders. This review sought to suggest which assessment tools would best clinically assess behavior in individuals with PMS. Methods: Validated behavioral assessment tools were identified using a systematic search of the literature, and relevant data for each assessment were extracted. The consensus guidelines for PMS were reviewed. Results: This review identified 131 validated assessment tools that were categorized by the intended age group and into specific behavioral domains: Autism spectrum disorder (ASD) screening, adaptive behavior, restricted and repetitive behaviors, challenging/disruptive behaviors, mental health screening, and other miscellaneous behaviors such as avoidance and impulsivity. Discussion: Based on the 2023 consensus guidelines, suggestions were given on which tools would be best for assessing various symptoms and behaviors in PMS. Choosing the best assessment tools to appraise behavior and related symptoms in individuals with PMS will aid clinicians in decision-making and lead to more personalized treatment plans.

## 1. Introduction

Phelan–McDermid Syndrome (PMS) is a rare neurodevelopmental disorder with phenotypic variability characterized by developmental delays (DDs), moderate to severe intellectual disability (ID), absent or delayed speech, seizures, sleep disturbances, gastrointestinal issues, minor dysmorphic traits, and a widespread array of behavioral symptoms, including ASD diagnoses in 50–60% of cases [1,2,3,4]. The genetic cause of PMS also varies with numerous terminal deletions in the 22q13.3 region, *SHANK3* haploinsufficiency, or pathogenic/likely pathogenic variants in *SHANK3*, which codes for the homonymous protein. SHANK3 is a synaptic scaffolding protein in the brain, and haploinsufficiency of *SHANK3* is considered the primary causative gene for the phenotype of PMS [3,4]. PMS, in part due to the heterogeneity of both genotype and phenotype, lack of evidence-based recommendations, and need for genotype–phenotype research, is considered underdiagnosed and challenging to assess clinically [1,4,5].

Common behavioral issues present in individuals with PMS include ASD or ASD-related behaviors, restricted and repetitive behaviors (RRBs), resistance to change, issues with socialization and communication, low levels of adaptive behavior needed for daily functioning, and self-injurious behavior, among other behavioral symptoms [1,2,4,5,6,7,8,9,10,11,12]. Other co-occurring diagnoses present in individuals with PMS include severe to profound ID, sensory sensitivities, aggression, anxiety, attention-deficit/hyperactivity disorder (ADHD), impulsivity, and other psychiatric diagnoses [1,3,4,12,13,14,15]. Behavioral issues have also been shown to increase stress and anxiety among parents and caregivers of individuals with PMS, potentially leading to familial distress [7,16].

While behavioral issues are common in PMS, numerous challenges currently limit the ability to accurately assess, diagnose, and treat behavior in affected individuals. The lack of knowledge surrounding behavior in PMS is partly due to the difficulty of assessing behavior in this population. While there is an abundance of behavioral assessment tools available, most are not intended for the PMS population or valid for use in individuals with ID, but instead targeted toward ASD, which may hinder the accuracy of behavioral assessments for individuals with PMS [1,12,17,18,19]. The level of ID present in an individual can increase the difficulty when assessing behavior and should be considered when choosing which assessment tool to utilize. Numerous calls have been made for more objective and precise assessment tools combined with direct observation to help accurately assess behavior in PMS [16,20,21].

A new set of clinical guidelines was recently published to provide the most up-to-date knowledge of PMS and guide clinicians, researchers, and families of individuals with PMS [13]. Additionally, a set of consensus recommendations was published through numerous individual publications in 2023 by the European PMS consortium to provide clinical recommendations for the care of individuals with PMS [22]. Both sets of guidelines involved input from experts in the field and parents of individuals with PMS to create a cohesive set of strategies to help aid clinical care. The guidelines offer in-depth advice for clinical assessments and management of all aspects of PMS; however, most of the advice calls for individuals to be routinely monitored and assessed for behavioral issues and related co-occurring symptoms without specifying which assessment tools should be utilized. As clinicians might feel some discomfort or inadequacy when treating patients with ID or neurodevelopmental disorders [23], having specific suggestions regarding which assessment tools to use in these populations would help clinicians adequately assess, diagnose, and treat individuals with ID, such as those with PMS. Due to the high prevalence of behavioral issues and related co-occurring diagnoses in individuals with PMS, the difficulties in accurately assessing behavior in this population, and the need for a deeper understanding of behavior in PMS, this study sought to review the literature to find validated behavioral assessment tools and use the recently published consensus guidelines on PMS to make suggestions as to which tools are available to assess behavioral issues in individuals with PMS in a clinical or research setting. Moreover, due to the diversity of the neurobehavioral phenotypes presented by individuals with PMS, this review may offer valuable insight for the behavioral assessment of other disorders that might share some features with PMS or might pose similar challenges due to their complex phenotypes.

## 2. Methodology

### 2.1. Literature Review

A systematic search and review of the literature was performed in PubMed, Web of Science, and Scopus. The literature search process was based on the 2020 PRISMA Guidelines [24], with the PRISMA-S for Reporting Literature Searches [25] utilized for the systematic search of the literature (Supplement S1). The search strategy was created in PubMed with key terms for behavior, ASD, assessment tools, and relevant MeSH terms (Table 1). The initial search strategy was then transformed for Web of Science and Scopus through Polyglot, a search translator [26].

**Figure 1:**
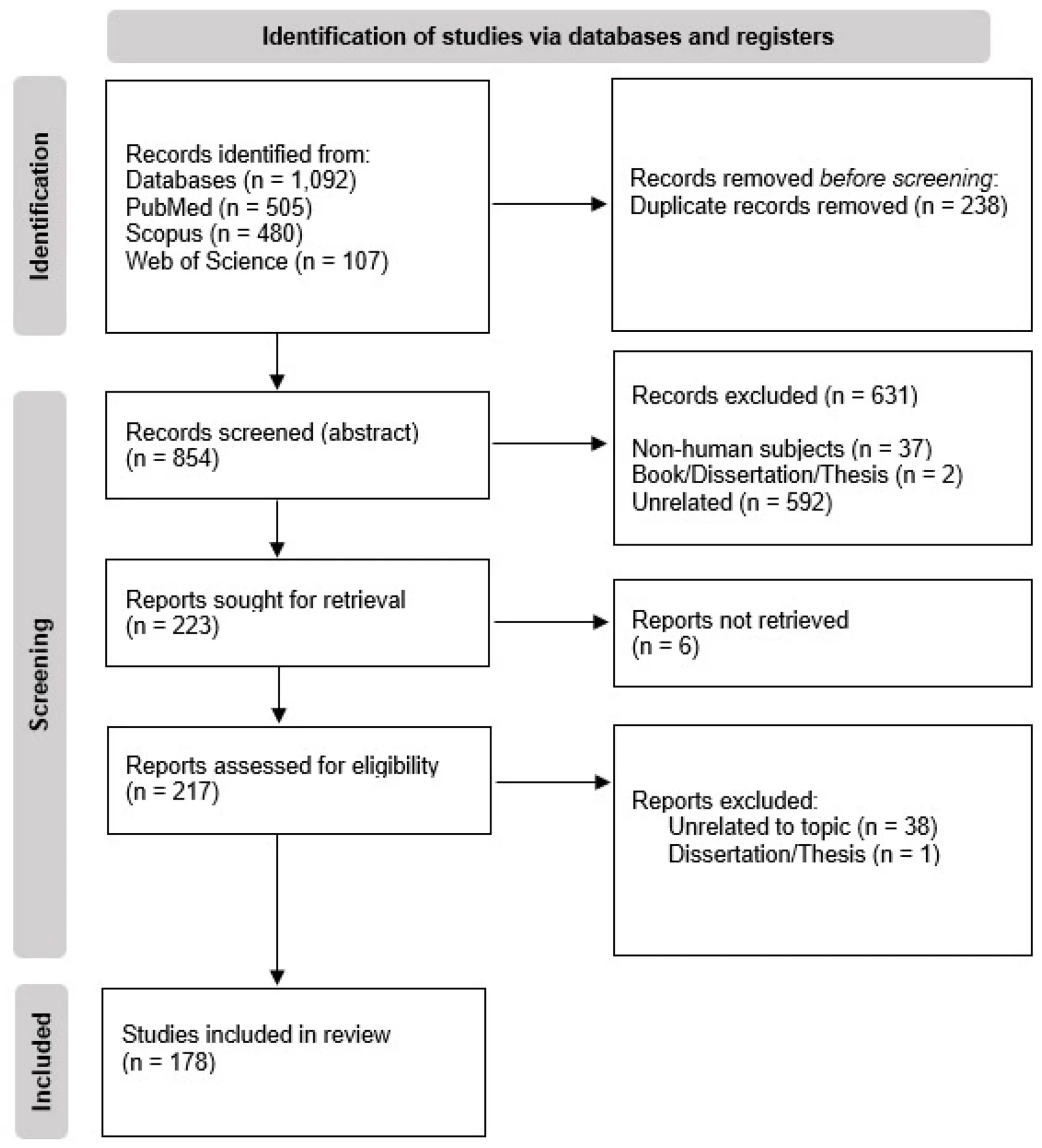
PRISMA 2020 flow diagram.

The initial search strategy between the three databases for the last ten years (1 January 2014–24 June 2024) resulted in 1092 articles imported into RefWorks, a citation management software. The 10-year cutoff ensured the search yielded assessment tools currently utilized in a research or clinical setting. Figure 1 outlines the article identification and screening process using the 2020 PRISMA flow diagram. Following the removal of 238 duplicate articles, 854 abstracts were screened for relevancy. During the abstract screening, 631 articles were excluded for either being performed on non-human subjects (animal or organoid), being from a book, dissertation, or thesis, or being irrelevant to the review. Of the remaining 223 articles, 217 articles were successfully retrieved for a full-text review using set inclusion and exclusion criteria. The set criteria included relevance to this review and full-text availability in English. Full-text articles were excluded for either being a dissertation/thesis or when found to be unrelated to the purpose of this review. 178 articles remained for subsequent data extraction to be performed by a single author. The data points extracted from each article included the behavioral assessment tools utilized, study population demographics, if available, and whether a specific disorder or symptom type was being studied.

### 2.2. Assessment Tool Review

Following the initial data extraction, a secondary data search and extraction process was performed for each assessment tool identified by reviewing the original reference source cited from the initial data extraction. Data points extracted, if available, included the year of origin, available versions, languages, purpose, intended age range, intended sample population or disorder type, intended level of cognitive ability, intended method of administration, number of items, average time for completion, and the specific domains or categories assessed.

### 2.3. Consensus Review

Finally, the PMS consensus guidelines [13] and consensus recommendations [14,15,22,27,28,29,30,31,32,33] were reviewed for the recent recommendations, guidance, and updated knowledge from clinical experts and parents regarding PMS. The up-to-date descriptions of PMS and recommendations for clinicians and researchers were utilized as the basis for this review’s suggestions regarding the choice of behavioral assessments for individuals with PMS.

The recommendations are based on a combination of various factors: psychometric performance, clinical utility, frequency of use, expert opinion, accessibility, and, most importantly, whether the tool was recommended and/or intended for use in ID/DD populations.

## 3. Results

### Literature Review

After a full-text review of 178 articles (Supplement S2), 131 validated behavioral assessment tools were identified. Following the assessment tool data extraction process (Supplement S3), the assessment tools were categorized into groups based on demographic characteristics and the behaviors the tool measured. Demographic characteristics included age and the presence of ID. The behavioral groupings were ASD screenings, adaptive behavior, restricted and repetitive behaviors (RRBs), disruptive/challenging behaviors, mental health, and a miscellaneous group including impulsivity, participation, cognition, and development, among others. Table 2 highlights each group of assessment tools and how many tools were identified per category. Table 3 shows the assessment tools utilized in five or more articles reviewed, and basic characteristics are also described for the top five most used tools (Table 3). While 131 assessment tools were identified, only 16 were used in five or more articles, highlighting the overabundance of available behavioral assessments but the skewed utilization of specific tools over others (Table 3). Some of the assessment tools utilized in other PMS studies include the ADI-R, ADOS, VABS, RBS-R, ABC, CBCL, SRS, and SP, which all require varying levels of qualifications and training to administer [1,8,21].

**Figure 2:**
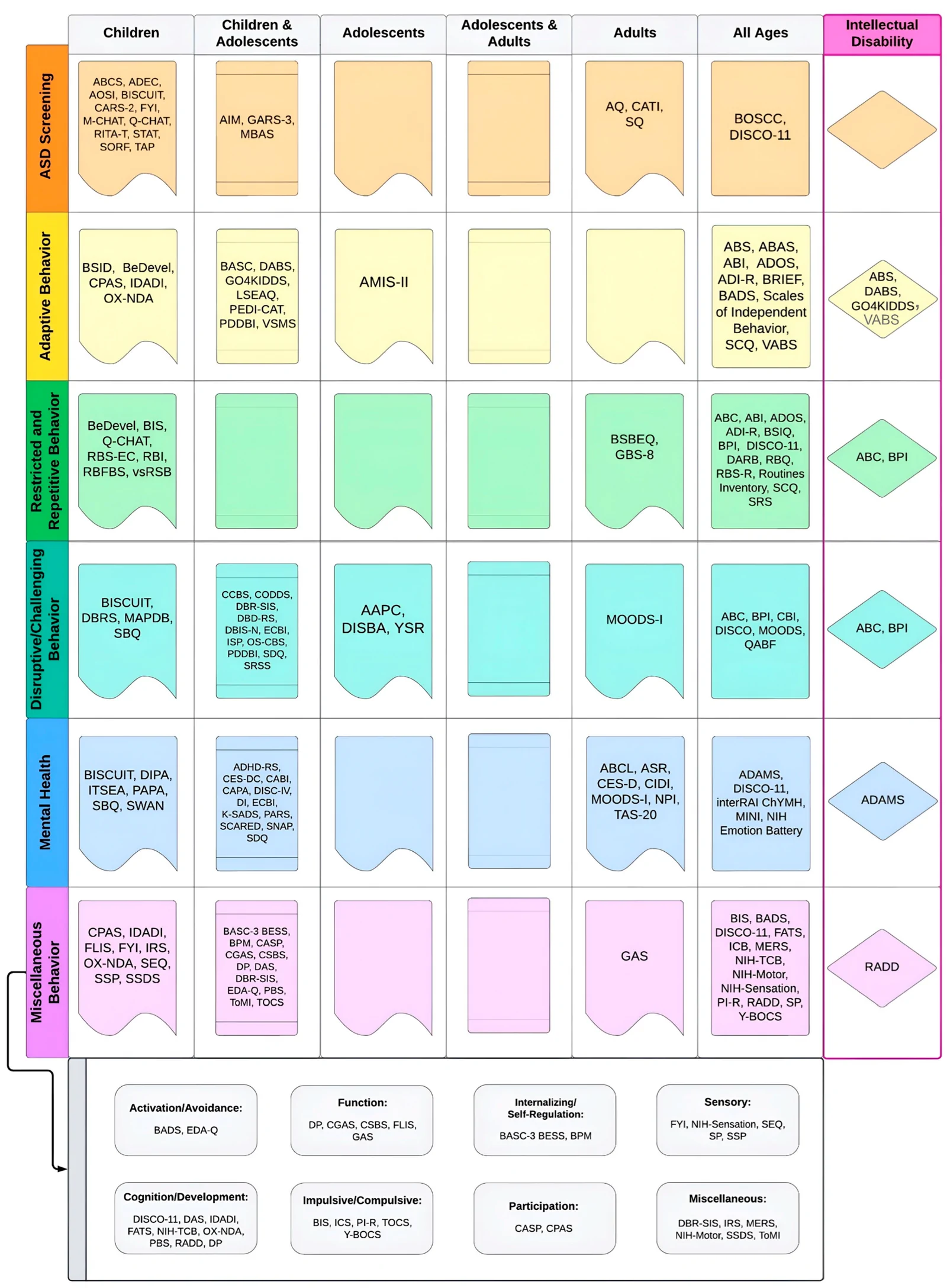
Behavioral assessment tools categorized by age, ID, and characteristic being assessed.

Figure 2 shows each identified assessment tool categorized by age group and type of behavior being assessed, including a column of tools specifically intended for use in individuals with ID. The abbreviation for each tool is used in the figure, but the full names of the assessment tools can be found in Supplement S4. This figure helps to quickly identify which tools might be the best options to assess a specific individual based on age, behavior type, and presence of ID.

**Figure 3:**
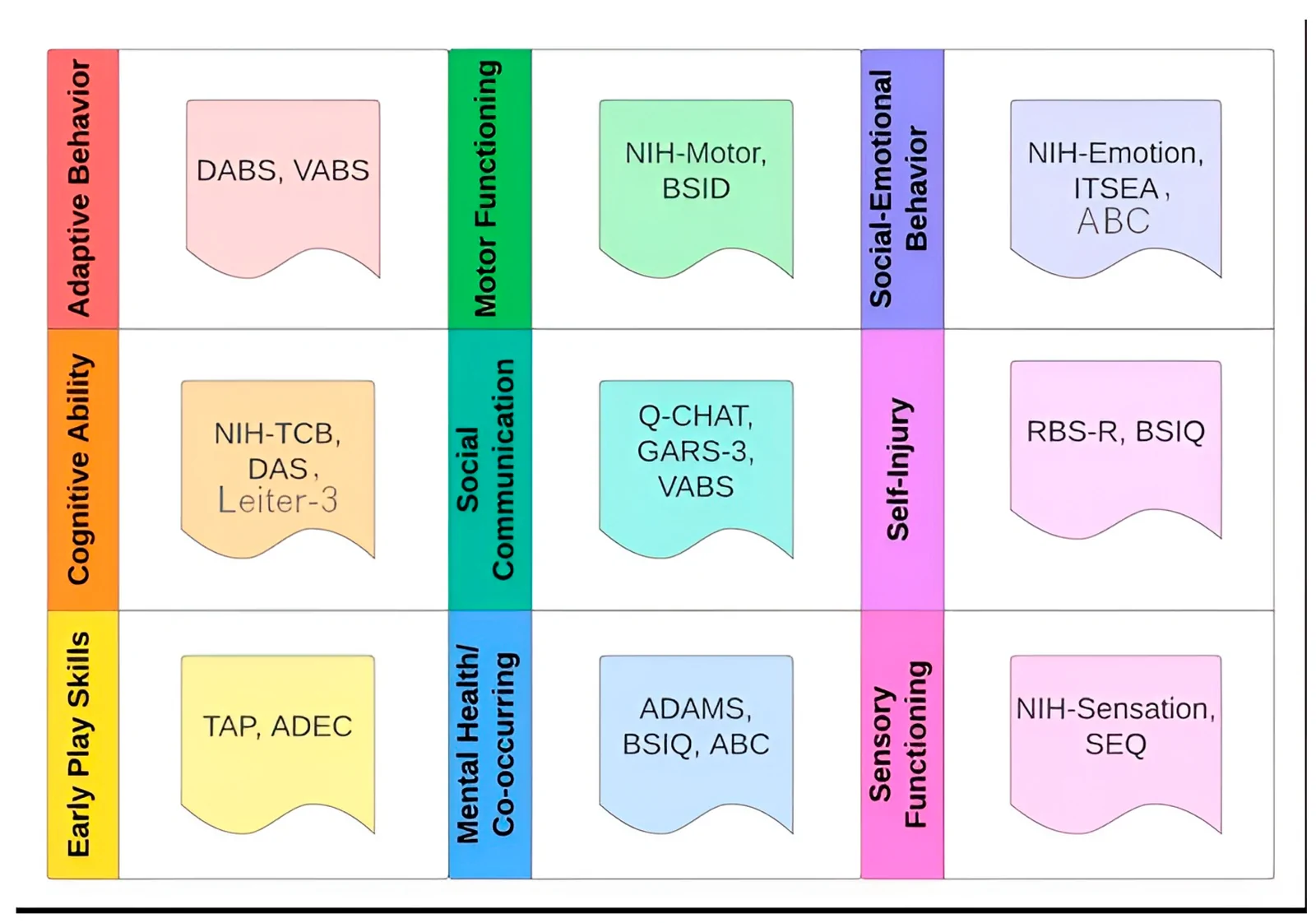
Suggested behavioral assessment tools categorized by characteristic being assessed.

Although ID/DD is described in 98% of individuals with PMS [2], only 8/131 (6.1%) of the assessment tools discussed in this study have been validated in subjects presenting with ID, as shown in Figure 2: ABS, DABS, GO4KIDDS (used to assess adaptive behavior), ABS and BPI (used for both RRBs and disruptive/challenging behaviors), ADAMS (mental health), and RADD (miscellaneous behavior). On the other hand, all the tools that we recommend for the assessment of behavioral issues have been validated primarily in individuals with ASD (see Figure 2 and Figure 3 for details). No assessment tool has been validated specifically in PMS, mostly due to the variability of the neurobehavioral phenotype in this condition.

## 4. Discussion

Figure 3 below highlights the main suggestions made throughout the discussion section based on the information from the consensus guidelines and assessments of each assessment tool. The recommendations are calibrated for the assessment of disorders affecting nine major neurobehavioral domains in individuals with PMS: adaptive behavior, motor functioning, social–emotional behavior, cognitive ability, social communication, self-injury, early play skills, mental health/co-occurring symptoms, and sensory functioning. However, it is important to consider that the severity and time of onset of disorders in each of these domains may vary. At the same time, behavioral phenotypes evolve across the lifespan, and therefore longitudinal assessment strategies may require different instruments at different developmental stages, as indicated in Figure 2. Moreover, whenever newer editions or revisions of the assessment tools are available, they should generally be preferred in clinical practice.

### 4.1. Suggestions Based on the American Consensus Guidelines

In the guidelines published by Srivastava et al., descriptions of behaviors in PMS are given along with related co-occurring diagnoses, followed by recommendations for future care and management in individuals with PMS [13].

Adaptive behavior

The guideline describes that in individuals with PMS, there tend to be low levels of adaptive behavior skills, the potential for regressions in self-help skills, RRBs, symptoms of sensory reactivity, and varying ID levels [13]. Based on this, Srivastava et al. [13] recommend routine monitoring of adaptive behavioral skills. Possible tools specifically intended for use with individuals with ID that measure adaptive behavior include the Adaptive Behavior Scale (ABS) [34], the Diagnostic Adaptive Behavior Scale (DABS) [35], the Vineland Adaptive Behavior Scales (VABS) [36], and Great Outcomes for Kids Impacted by Severe Developmental Disabilities Brief Adaptive Behavior Scale (GO4KIDDS) [37]. The ABS, DABS, and VABS are available for individuals of all ages, but the GO4KIDDS is intended only for children and adolescents. The DABS and VABS are widely used in research and clinical settings compared to the ABS and GO4KIDDS, which are not often used clinically. The DABS and VABS are readily available for purchase, with the DABS being substantially less expensive than the VABS. The VABS requires a trained interviewer to perform the assessment, while the DABS offers optional training. The DABS and VABS take roughly an hour to complete, but the VABS is flexible, and not all questions must be answered to complete the assessment.

Cognitive ability

It is also recommended that routine monitoring of cognitive skills is performed and early play skills in young children evaluated [13]. Numerous validated tools monitor cognitive skills, such as the Differential Ability Scales (DAS) [38] and the NIH Toolbox Cognitive Battery (NIH-TCB) [39]. An additional assessment tool that did not result during the literature review, but that can be used to assess cognitive skills in a population with ID is the Leiter-3, which is a non-verbal measure of intelligence for individuals three years old and above [40]. The non-verbal aspect of the Leiter-3 makes it particularly useful in a population such as PMS. The Rapid Assessment for Developmental Disabilities (RADD) [41] assesses cognitive ability and is specifically intended for individuals with developmental disabilities, but appears to be challenging to access. The DAS and Leiter-3 require a qualified psychologist to administer, whereas the NIH-TCB is readily available through a downloadable application for purchase and then requires a psychologist to interpret the results.

Early play skills

Assessments to evaluate early play skills include the TELE-ASD-PEDS (TAP) [42], Autism Detection in Early Childhood (ADEC) [43], and Behavior Development Screening for Toddlers (BeDevel) [44]. The BeDevel was intended for use in Korean populations only and, therefore, might not be easily accessible or applicable to all populations. The TAP and ADEC are intended for use in clinical and research settings. The TAP is easily accessible, allows for remote observation by a provider, and can be completed in under 20 min. The ADEC is available for purchase and can be completed by either a provider or with provider oversight in under 15 min.

Motor functioning and social communication

The guidelines recommend monitoring related difficulties such as motor functioning and social communication [13]. The Autism Observation Scale for Infants (AOSI) [45] in toddlers or the Dimensional Inventory for Child Development Assessment (IDADI) [46] can assess both motor functioning and social communication. However, the IDADI was intended for use in the Brazilian culture and might not apply to larger populations. The Bayley Scale of Infant and Toddler Development (BSID) [47] also measures motor functioning in younger children and toddlers in either a clinical or research setting within an hour or less. The BSID is available for purchase and requires some training to utilize. The NIH Toolbox Motor Battery (NIH-Motor) [39] can assess motor functioning in individuals of all ages through a downloadable application for purchase, which might be best used for continuous monitoring. Social communication can be assessed in toddlers through various tools such as the Quantitative Checklist for Autism in Toddlers (Q-CHAT) [48], First Year Inventory (FYI) [49], and Behavior Development Screening for Toddlers (BeDevel) [44]. Additionally, the Gilliam Autism Rating Scale (GARS-3) [50] can be used for children and adolescents through the early twenties, and the Autism Diagnostic Observation Schedule (ADOS) can be used for all ages [51]. Of these, the Q-CHAT and GARS-3 are the best candidates. First, both tools are meant for research and clinical settings, unlike the FYI, and can be completed in around 10 min. The GARS-3 can be performed with in-person assessments or with a parental report, and the Q-CHAT is also completed through a parental report. The GARS-3 and the Q-CHAT are more easily accessible, whereas the ADOS requires a trained clinician and typically requires a referral for access.

Mental health and co-occurring symptoms

The consensus guidelines also describe various co-occurring symptoms related to behavioral issues. Some of these include symptoms related to depression, mood disorders, ADHD, aggression, and anxiety [13]. A wide variety of tools is available to assess these related symptoms across all age groups; however, some may be better suited for use in individuals with PMS. A large number of tools exist for depression, ADHD, and anxiety in children and adolescents that are not intended or validated for use with individuals with ID or other neurodevelopmental disorders, such as the Pediatric Behavior Scale (PBS) [52] and Dominic Interactive (DI) [53]. The Anxiety Depression and Mood Scale (ADAMS) [54] can assess depression, mood disorders, ADHD, and anxiety in all ages and is intended for use in individuals with ID. The Behavior and Sensory Interests Questionnaire (BSIQ) [55] can also assess aggression in individuals 18 months or older with neurodevelopmental disorders. The Aberrant Behavior Checklist (ABC) [56] can assess behavior and psychiatric problems in individuals with ID for all ages above five years old. Therefore, tools such as the ADAMS, ABC, and BSIQ are more applicable to individuals with PMS when assessing co-occurring diagnoses such as depression, anxiety, aggression, and mood disorders. The BSIQ requires training to administer and takes around 45 min, while the ABC and ADAMS require minimal training and can be completed in around 15 min.

### 4.2. Suggestions Based on the European Consensus Recommendations

In the consensus recommendations published by the European PMS consortium, each publication focuses on a specific symptom or related issue in individuals with PMS and details the symptoms while giving recommendations for care and management [22]. The recommendations state that not only can difficulties in communication lead to behavioral issues, but the behavioral issues found in PMS are inherent to the diagnoses of ID, mood disorders, and ASD [27]. They suggest that individuals with PMS should be evaluated for various factors influencing communication. Therefore, as previously mentioned, tools such as the VABS [36] or DABs [35] for adaptive behavior and the ADAMS [54] for mood disorders could be used to assess these symptoms. For social–emotional behaviors, various tools exist, but the Infant Toddler Social–Emotional Assessment (ITSEA) [57], the Learning, Social, and Emotional Adaptation Questionnaire (LSEAQ) [58], the Aberrant Behavior Checklist (ABC) [56], or the NIH Toolbox Emotion Battery (NIH-Emotion) [39] assessments might be best served in PMS because the ITSEA is intended for toddlers with developmental disorders, the LSEAQ is for children/adolescents with ASD or ASD-related disorders, the ABC can be utilized for anybody over the age of five years old with ID, and the NIH-Emotion assesses social–emotional behaviors across all ages. While the LSEAQ is mainly used in a school setting, the NIH-Emotion, ABC, and ITSEA are used in research and clinical settings. Both the ITSEA and NIH-Emotion have training available to complete, while the NIH-Emotion is more accessible through a downloadable application for purchase. The ABC requires minimal training and is available for purchase.

Additionally, symptoms such as behavioral issues, a loss of adaptive skills, and self-injurious behaviors can be signs of underlying health issues such as gastrointestinal disorders, lymphedema, altered sensory functions, or the presence of pain [15,28,31]. Assessing the behavioral issues in PMS could help determine other underlying health issues. Adaptive behaviors can be assessed with the same tools previously described. Self-injurious behaviors can be assessed using multiple tools, such as the Repetitive Behavior Scale-Revised (RBS-R) [59] and BSIQ [55], which are intended for all age groups and individuals with ASD; the BSIQ is also intended for individuals with neurodevelopmental disorders. The RBS-R requires minimal training, is easily accessible, and can be completed in under 15 min, while the BSIQ is lengthier and requires more training.

Co-occurring ASD, ADHD, anxiety, or depression can increase sleep disturbances and should be investigated to help with sleep disturbances commonly described in individuals with PMS [32]. As previously mentioned, the ADAMS [54] is intended to assess symptoms, such as inattention, hyperactivity, and anxiety, in ID across all age groups and might be a helpful tool to evaluate potential symptoms associated with sleep disturbances. Changes in behavior and anxiety can also indicate altered sensory functioning and atypical responses to sensory stimuli, so Walinga et al. recommend the Short Sensory Profile (SSP) to assess sensory issues in individuals with PMS [15]. However, the SSP [60] is intended for ages 3–10, while other tools to assess sensory issues, such as the NIH Toolbox Sensation Battery (NIH-Sensation) [39], can be utilized across all ages. Additionally, the Sensory Experiences Questionnaire (SEQ) [61] is intended for individuals with neurodevelopmental disorders, which might be better suited for individuals with PMS. The SEQ can be completed in under 20 min with no or minimal training needed. The NIH-Sensation can be completed electronically by purchasing a downloadable application with training available, and both tools are meant for use in clinical and research settings.

Mental health issues are common in individuals with PMS, including anxiety, avoidance, and aggression, along with other behavioral issues such as self-injury, RRBs, and loss of adaptive behavior skills [14]. The recommendations by Van Balkom et al. include numerous domains of behavior and co-occurring symptoms that need to be assessed in PMS, such as general functioning, development, communication, mood, adaptive functioning, and mental health issues [14]. The assessment tools previously mentioned for adaptive behavior, mental health, and sensory functioning should be prioritized for these assessments in individuals with PMS. The Diagnostic Instrument for Social and Communication Disorders (DISCO-11) [62] is intended for ASD in all age groups to assess development. However, it takes a few hours to complete and requires a trained clinician to administer. Tools such as the GO4KIDDS [37] and VABS [36] could be used to assess communication because they are both intended and validated for use in individuals with developmental disabilities; however, the VABS can be used in both research and clinical settings, while the GO4KIDDS is used primarily in research settings. Avoidance can be assessed using the ADAMS [54] for all ages or the ABC [56] for anybody over five years old, which are both validated for individuals with ID, commonly used in both research and clinical settings, and completed in under 15 min with no or minimal training needed.

### 4.3. Limitations

One of the primary aims of this review was to create a comprehensive list of validated behavioral assessment tools used in a research or clinical setting. While the review yielded a list of 131 assessment tools, this list cannot be seen as exhaustive, and some assessment tools could have been missed. However, with an initial starting point of 1092 articles and, ultimately, 178 full-text reviews of relevant articles, the review was extensive and should have identified most of the available assessment tools. Additionally, this review limited the search of articles to the last ten years, so not all assessment tools might have been found through the limited time period. However, the intention of limiting to the last ten years was to find assessment tools that are relatively current and being used in either a research or clinical setting. Moreover, data extraction was performed by a single reviewer, and only scientific articles written in English were included.

The other primary aim of this review was to provide suggestions for assessing behavior in individuals with PMS based on information found in the 2023 PMS consensus guidelines [13] and the 2023 consensus recommendations from the European PMS consortium [14,15,22,27,28,29,30,31,32,33]. The guidelines do not explicitly list tools that should be used, so the information given in the guidelines was used to help suggest potential assessments to measure behavior in individuals with PMS. It is also important to underline that most recommended instruments have not been specifically validated in individuals with PMS. While the suggestions from this review have an element of subjectivity, their basis relies on an extensive review of the recent literature and recommendations from the most up-to-date information published for PMS by experts in the field. Finally, the striking variability in the neurobehavioral phenotypes reported in people with PMS suggests that recommendations like the ones proposed in this review must be filtered by specialized healthcare professionals while considering the unique profile of each individual affected by PMS.

## 5. Conclusions

One of the pillars of precision medicine is the utilization of standardized, validated protocols to assess signs and symptoms and collect clinical data to correlate and integrate with instrumental and laboratory information. The ultimate goal is to develop multidisciplinary approaches to refine the clinical, genetic, and functional profiles of individual patients in order to provide the most effective treatments. PMS poses numerous challenges both in terms of clinical variability and genetic heterogeneity. While a vast array of validated behavioral assessment tools is available, there might still be difficulties when assessing behavior in individuals with PMS. This is mainly due to the common presence of communication difficulties, severe to profound levels of ID in PMS, and the lack of assessment tools specifically designed to assess individuals with ID and difficulties communicating. Only a handful of the tools identified in this review specifically state the ability to assess individuals with ID, and even these tools can fall victim to floor effects and unreliable results in an ID population. In contrast, many others still need to be adequately validated in this population before being used as a reliable assessment. Future research must validate assessment tools in behavioral domains such as adaptive behavior, RRBs, challenging behavior, and miscellaneous behaviors such as obsessive–compulsive behavior, sensory sensitivities, and cognitive and developmental delays. Additionally, this review revealed a gap in behavioral assessment tools specific to the adult population, with a larger focus on children and adolescent populations, which could hinder the continued assessment of adult individuals with PMS. This overabundance might be overwhelming and confusing when determining the best way to assess behavior clinically. Instead of creating new assessment tools to add to the growing list, an effort should be made to take pre-existing tools, update them as needed, and validate them for use in various populations, such as ID, ASD, and other neurodevelopmental disorders, such as PMS.

## 6. Future Directions

Multiple barriers might prevent access to the recommended assessment tools for individuals with PMS. These barriers include difficulties accessing care from trained clinicians and psychologists and challenges with billing and insurance coverage. Some of these assessment tools (i.e., VABS) require licensing costs that may limit accessibility for physicians operating in low-resource settings. Outcomes from other tools may be influenced by factors like cross-cultural availability, translations, or social conventions and background: this bias may apply, for example, to the GARS-3, the Q-CHAT, the ADOS, and, in general, any assessment tool based at least in part on parental reports.

Efforts should be made in the future to allow decreased costs and better insurance coverage for access to the recommended assessment tools. More effort should be taken to help make medical care and assessment tools more accessible through the use of telehealth and telepsychology platforms in the future. These future efforts would decrease the confusion surrounding assessing behavior in individuals with PMS. In the meantime, this review can help point clinicians, researchers, and parents in the best direction to assess behaviors in PMS accurately and correlate them more precisely with genetic alterations, leading to advancing our knowledge on the etiology of specific behavioral disorders. Additionally, when behavior and other related symptoms are accurately assessed, this will help lead toward more individualized and specific plans of care, in line with the scope of precision medicine. Proper assessment of individuals with PMS will ultimately increase the confidence of clinicians working with individuals with PMS, improve the level of care the clinician can provide, generate more knowledge about behavior in PMS, and potentially lead to more specific interventions.

## Figures and Tables

**Table 1:** Literature Review Search Strategy by Database. Caption: Systematic search strategy of this review for each database utilized.

Database	Search Strategy
**PubMed**	(“restrictive behav*”[All Fields] OR “repetitive behav*”[All Fields] OR “restrictive repetitive behav*”[All Fields] OR “RRBs”[All Fields] OR “RRB”[All Fields] OR “restricted repetitive behav*”[All Fields] OR “adaptive behav*”[All Fields] OR “challenging behav*”[All Fields] OR “disruptive behav*”[All Fields] OR “behavior issue*”[All Fields] OR “behaviour issue*”[All Fields] OR “autism like behav*”[All Fields]) AND (“assess”[All Fields] OR “assessed”[All Fields] OR “assessment”[All Fields] OR “assesses”[All Fields] OR “assessing”[All Fields] OR “assessment”[All Fields] OR “assessment s”[All Fields] OR “assessments”[All Fields] OR “tool”[All Fields] OR “screen*”[All Fields] OR (“guideline”[Publication Type] OR “guidelines as topic”[MeSH Terms] OR “guideline”[All Fields]) OR (“instrument”[All Fields] OR “instrument s”[All Fields] OR “instrumentation”[MeSH Subheading] OR “instrumentation”[All Fields] OR “instruments”[All Fields] OR “instrumented”[All Fields] OR “instrumenting”[All Fields])) AND “valid*”[All Fields] AND (“human s”[All Fields] OR “humans”[MeSH Terms] OR “humans”[All Fields] OR “human”[All Fields])
**Web of Science**	(AB=“restrictive behav*” OR AB=“repetitive behav*” OR AB=“restrictive repetitive behav*” OR AB=RRBs OR AB=RRB OR AB=“restricted repetitive behav*” OR AB=“adaptive behav*” OR AB=“challenging behav*” OR AB=“disruptive behav*” OR AB=“behavior issue*” OR AB=“behaviour issue*” OR AB=“autism like behav*”) AND (AB=measure* OR (AB=assess OR AB=assessed OR AB=assessement OR AB=assesses OR AB=assessing OR AB=assessment OR AB=“assessments” OR AB=assessmenAB) OR AB=tool OR AB=screen* OR (AB=guideline OR AB=“guidelines as topic” OR AB=guideline) OR (AB=instrument OR AB=“instrument s” OR AB=instrumentation OR AB=instrumentation OR AB=instrumenAB OR AB=instrumented OR AB=instrumenting)) AND AB=valid* AND (AB=“human s” OR AB=humans OR AB=humans OR AB=human)
**Scopus**	(ABS(“restrictive behav*”) OR ABS(“repetitive behav*”) OR ABS(“restrictive repetitive behav*”) OR ABS(RRBs) OR ABS(RRB) OR ABS(“restricted repetitive behav*”) OR ABS(“adaptive behav*”) OR ABS(“challenging behav*”) OR ABS(“disruptive behav*”) OR ABS(“behavior issue*”) OR ABS(“behaviour issue*”) OR ABS(“autism like behav*”)) AND (ABS(measure*) OR (ABS(assess) OR ABS(assessed) OR ABS(assessement) OR ABS(assesses) OR ABS(assessing) OR ABS(assessment) OR ABS(“assessment s”) OR ABS(assessments)) OR ABS(tool) OR ABS(screen*) OR (DOCTYPE(guideline) OR INDEXTERMS(“guidelines as topic”) OR ABS(guideline)) OR (ABS(instrument) OR ABS(“instrument s”) OR INDEXTERMS(instrumentation) OR ABS(instrumentation) OR ABS(instruments) OR ABS(instrumented) OR ABS(instrumenting))) AND ABS(valid*) AND (ABS(“human s”) OR INDEXTERMS(humans) OR ABS(humans) OR ABS(human))

**Table 2:** Number of Assessment Tools Identified per Intended Study Group and Intended Target Variable. * Each tool can be found in more than one category, so the total will not equal 131. Caption: Breakdown of each included assessment tool identified during the review, categorized by the intended study group and the targeted variable or characteristic of each assessment tool.

**Intended Study Group**	Number of Assessment Tools Identified *
**Children**	75
**Adolescent**	43
**Adult**	15
**All ages**	37
**Intellectual disability (ID)**	8
**Intended Target Variable**	Number of Assessment Tools Identified *
**General ASD screening**	20
**Adaptive behavior**	23
**Restricted and repetitive behavior (RRB)**	22
**Disruptive/challenging behavior**	25
**Mental health**	30
General mental health	18
Attention-deficit/hyperactivity disorder (ADHD)	9
Mood/Depression	11
Anxiety	11
Alexithymia	1
Miscellaneous behavior *	35
Activation/avoidance	2
Cognition/development	9
Functioning	5
Impulsive/compulsive	5
Internalizing/self-regulation	2
Participation	2
Sensory	5
Miscellaneous	6

**Table 3:** Assessment Tools Utilized in Five or More Reviewed Articles and Basic Characteristics for the Top Five Most Utilized Tools. Caption: List of assessment tools that were identified in five or more of the reviewed articles, with basic characteristics included for the top five most utilized assessment tools.

Assessment Tool					Number of Articles	Assessment Tool (Continued)	Number of Articles (Continued)
**Vineland Adaptive Behavior Scale (VABS)**					47	**Aberrant Behavior Checklist (ABC)**	10
0–90+ years	intended for ID & DD	parent or caregiver interview or self-report	502 items	<1 h			
**Autism Diagnostic Observation Schedule (ADOS)**					25	**Adaptive Behavior System (ABAS)**	9
all ages	intended for suspected ASD	standardized interviewer	93 items	1.5–2.5 h			
**Social Responsiveness Scale (SRS)**					21	**Childhood Autism Rating Scale (CARS)**	8
>2.5 years	intended for ASD	parent or caregiver or self-report	65 items	20 min			
**Child Behavior Checklist (CBCL)**					21	**Disruptive Behavior Disorder Rating Scale (DBD-RS)**	7
children & adolescents		parent report	99–113 items	15–20 min			
**Repetitive Behavior Scale-Revised (RBS-R)**					19	**Multidimensional Assessment Profile of Disruptive Behavior (MAPDB)**	6
2–62 years	intended for ASD	caregiver or self-report	43 items	15 min			
**Social Communication Scale (SCQ)**					17	**Repetitive Behavior Questionnaire (RBQ)**	6
**Autism Diagnostic Interview-Revised (ADI-R)**					13	**Kiddie-Schedule for Affective Disorders and Schizophrenia for School-Age Children (K-SADS)**	5
**Strengths and Difficulties Questionnaire (SDQ)**					11	**Eyberg Child Behavior Inventory (ECBI)**	5

## Data Availability

The authors confirm that the data supporting the findings of this study are available within the article [and/or] its Supplementary Materials.

## Supplementary Materials

The following supporting information can be downloaded at: https://www.mdpi.com/article/10.3390/genes17080923/s1. Supplement S1: PRISMA-S for Reporting Literature Searches; Supplement S2: Full list of articles; Supplement S3: Assessment tool data extraction process; Supplement S4: Full names of assessment tools.
